# The effectiveness of radial extracorporeal shock wave therapy (rESWT), sham-rESWT, standardised exercise programme or usual care for patients with plantar fasciopathy: study protocol for a double-blind, randomised, sham-controlled trial

**DOI:** 10.1186/s13063-020-04510-z

**Published:** 2020-06-29

**Authors:** Marte Heide, Marianne Mørk, Cecilie Røe, Jens Ivar Brox, Aasne Fenne Hoksrud

**Affiliations:** 1 Faculty of Medicine, University of Oslo, Postboks 1078, Blindern, Oslo, Norway; 2 Department of Physical Medicine and Rehabilitation, Oslo University Hospital, Postboks 4956, Ullevål, Nydalen, 0242 Oslo, Norway

**Keywords:** Plantar fasciopathy, Plantar fasciitis, Radial extracorporeal shock wave therapy, Sham-radial extracorporeal shock wave therapy, High-load strength training, Foot orthosis, Randomised controlled trial

## Abstract

**Background:**

Plantar fasciopathy is a common cause of plantar heel pain, with a reported prevalence of up to 10%. The choice of best practice in these patients is debated. Two randomised studies reported that radial extracorporeal shock wave therapy is effective, but a meta-analysis concluded that due to methodological limitations, the evidence is questionable. There are few studies reporting the effect of exercise programs with high-load strength training, despite widespread use. The objective of this placebo-controlled, observer-blinded and partly patient blinded trial is to compare rESWT, sham-rESWT, standardised exercise programme and usual care for alleviating heel pain at 6 and 12 months follow-up.

**Methods/design:**

A double-blind, randomised, sham-controlled trial is conducted at a hospital outpatient clinic of physical medicine and rehabilitation. Patients with chronic (> 3 months) pain due to plantar fasciopathy, aged 18 to 70 years old, are eligible for inclusion in the trial. Patients will be randomly allocated in 1:1 ratio to receive rESWT, sham-rESWT, standardised exercises or usual care. The sample size is estimated to 200 patients, 50 in each group. rESWT or sham-rESWT will be given once a week for 3 weeks. A physiotherapist will supervise the exercises, with a total of 8 sessions over 12 weeks. The patients in the usual care group will receive information, advice and foot orthosis only. All patients, regardless of group, will receive the same information and get an individual customised foot orthosis made by an orthopaedic technician. The primary outcome measure is heel pain intensity during activity in the last week, using a numeric rating scale (NRS, 0 to 10) at the 6 months follow-up adjusted for baseline pain intensity. The secondary outcomes are at the 6- and 12-month follow-up and include Foot Functional Index Revised Short Version (FFI-RS), Patient Global Impression of Change Scale (7-point Likert scale), RAND-12 Health Status Inventory (RAND-12), NRS during rest and NRS during activity (12 months). The patients receiving rESWT/sham-rESWT and the outcome assessor will be blinded to the group assignment.

**Discussion:**

This trial is designed in order to provide results important for future clinical practice.

**Trial registration:**

ClinicalTrials.gov NCT03472989. Registered on 14 March 2018

## Background

Plantar fasciopathy is a common cause of plantar heel pain in adults, with reported lifetime prevalence up to 10% [1, 2]. Plantar fasciopathy is often referred to as plantar fasciitis. Histological research indicates that plantar fasciitis is not an acute inflammation, but rather a degenerative process [3]. Based on this knowledge, we have chosen to use the term plantar fasciopathy. The condition seems to be gender invariant and affect both athletic and non-athletic individuals [4]. The prognosis for plantar fasciopathy is unclear. It is reported to resolve over time in most people [4–6] and that about 80% of patients will become symptom-free within 1 year regardless of the treatment they receive [1, 6], but this estimate varies. In a Danish cohort study by Hansen et al. [7], including patients with severe plantar fasciopathy, the risk of still having the condition was 50% after 5 years and 44% after 15 years from the onset of symptoms. The patients commonly report prolonged problems with standing, walking and running. It has been found to cause a reduction in physical activity [2, 8], health-related quality of life [2] and to be a financial burden on the community [9].

The diagnostic criterion for plantar fasciopathy is generally defined as pain at the medial tubercle of the calcaneus [1, 4], which may extend to the medial longitudinal arch of the foot [4]. Tenderness on palpation corresponding to the painful area is also generally required [5]. A typical symptom is pain at the first step after a period of rest. The pain often improves after a few steps, but may recur after prolonged, continued or more stressful activity [5, 6]. The diagnosis is primarily based on the patient’s history and clinical examination [10]. Imaging, like ultrasound, is usually not required in the diagnostic. The prognostic impact of typical findings on ultrasound such as increased fascial thickness (> 4 mm), hypoechogenicity and calcification within the plantar fascia and Doppler ultrasound-identified hyperaemia is uncertain [11].

The aetiology of plantar fasciopathy is still unclear [4]. It is assumed to develop due to biomechanical stress of the plantar fascia and its enthesis of the medial calcaneal tuberosity [5, 8]. High body mass index (BMI), decreased dorsiflexion of the first metatarsophalangeal joint, decreased ankle dorsiflexion, prolonged standing and increased age all demonstrate some association with plantar fasciopathy [12, 13].

Due to the unclear understanding of the aetiology, several different conservative therapeutic interventions have been recommended for the management of plantar fasciopathy, including corticosteroid injections, NSAIDs, night splints, taping, stretching, exercise, foot orthosis and extracorporeal shock wave therapy (ESWT) [4, 10]. There are promising results from randomised controlled trials (RCTs) investigating different treatment regimes in patients with plantar fasciopathy [4, 14], but to our knowledge, there is still no agreement on which treatment to recommend.

ESWT (both focused ESWT and radial ESWT/rESWT) has been used in the treatment of soft tissue and musculoskeletal disorders, including plantar fasciopathy, for many years [15, 16]. Of the patients referred to our outpatient clinic, an increasing number have tried ESWT. The radial shock wave systems are especially popular because of its applicability and lower costs [17]. The theories on the effects of ESWT include pain relief [15], destruction of calcification and stimulation of tissue regeneration [15, 18]. Two RCTs have investigated the effect of rESWT compared to sham-rESWT, demonstrating promising results [19, 20]. Gerdesmeyer et al. used three interventions of rESWT, while Ibrahim et al. used two interventions. Either of the studies reported the success of blinding. A meta-analysis concluded that due to methodological limitations, the evidence on the effectiveness of rESWT is questioned [16]. Our purpose is to include both rESWT and sham-rESWT in the present trial, to further investigate the effectiveness of this treatment and also evaluate the blinding success to evaluate performance bias.

There are studies with promising results on the effectiveness of exercise in plantar fasciopathy, but the studies have methodological limitations [21–23]. High-load strength training programs are often recommended in the treatment of tendinopathies, and there have been promising results regarding Achilles and patellar tendinopathy [24]. The plantar fascia is made up of predominantly longitudinally oriented collagen fibres, as in other tendons and ligaments [1]. A biomechanical experiment has demonstrated that dorsiflexion of the metatarsophalangeal joints in the foot tightens the plantar fascia. Together with a tensile force applied to the Achilles tendon, it creates high tensile loads across the plantar fascia [25]. This theory was used in a small study by Rathleff et al. from 2014, which compared strength training with unilateral heel raise using a towel inserted under the toes, and stretching of the plantar fascia. Pain reduction was better in the strength training group after 3 months, but not after 12 months [23]. In a recent study from Riel et al., a self-dosed and a pre-determined heavy slow resistance non-supervised training programme over 12 weeks was compared. The self-dosed training programme did not reduce pain more than the pre-determined programme. Both groups had improvement in pain larger than the minimum clinically important difference, but only a few in both groups achieved Patient Acceptable Symptom State. None of the groups had supervision by a physiotherapist during the 12 weeks to ensure the correct technique, and no long-term effect was measured (> 12 weeks) [26]. We have an impression that physiotherapists often try some form of a strength training programme in the treatment of patients with plantar fasciopathy. We believe there is a need for more high-quality studies investigating the effect of high-load strength training programs.

The natural course of a disease is often difficult to capture due to the ethical aspects of offering no treatment and the risk of a more selected patient population in such studies. The provision of usual care may reduce selection bias and improve relevance and external validity [27]. The usual care at our outpatient clinic includes information on the pathogenesis, aetiology and prognosis based on the latest research. We have experienced, as reported by Irving et al. [2], that some patients become inactive, which can lead to isolation and a depressed mood. We try to motivate the patient to be active and accept moderate pain during activity without becoming worried. We suggest an alternate activity if pain is the main limitation. We also recommend that patients use proper footwear including foot orthosis. Both prefabricated and custom-fitted orthoses are commonly recommended in the treatment of plantar fasciopathy [6]. Two similar systematic review and meta-analysis were recently published on the effectiveness of foot orthosis for plantar heel pain. Whittaker et al. found moderate-quality evidence that foot orthosis is more effective than sham orthosis to reduce pain, but it was unclear whether this was a clinically meaningful change [28]. Rasenberg et al. found no difference in improvement in pain or function when comparing prefabricated, sham orthosis or custom-made orthosis [29]. There was heterogeneity when comparing the included trials, leading to limited evidence.

In our trial, we will include patients with chronic symptoms (> 3 months). Plantar fasciopathy may have a prolonged course over years for some patients, and it will be of importance regarding the quality of life of the patients and also in a socioeconomic perspective to shorten the duration of symptoms. To our knowledge, there are no other previous trials comparing rESWT, sham-rESWT and exercises to usual care. This trial is designed in order to provide results important for future clinical practice.

## Research hypothesis

### Null hypothesis

H0: There is no difference between rESWT, sham-rESWT and a standardised exercise programme on change in heel pain (primary outcome) and functioning (secondary outcomes) compared to usual care in the treatment of plantar fasciopathy at 6 months follow-up (and secondary outcomes at 12 months follow-up).

### Alternative hypothesis

- H1: There is a difference between rESWT and usual care on change in heel pain (and secondary outcomes) at 6 months follow-up (and secondary outcomes at 12 months follow-up).
- H2: There is a difference between sham-rESWT and usual care on change in heel pain (and secondary outcomes) at 6 months follow-up (and secondary outcomes at 12 months follow-up).
- H3: There is a difference between the standardised exercise programme and usual care on change in heel pain (and secondary outcomes) at 6 months follow-up (and secondary outcomes at 12 months follow-up).

## Methods

### Design

This is a double-blind, randomised, sham-controlled trial, with four parallel groups. All patients will be recruited from the Department of Physical Medicine and Rehabilitation at Oslo University Hospital, Norway.

### Participants

Patients referred to the clinic with heel pain, aged between 18 and 70 years old, are eligible for inclusion.

The inclusion criteria are as follows: pain with duration over 3 months localised in the proximal insertion of the plantar fascia on the medial calcaneal tuberosity, and tenderness to palpation corresponding to the painful area. The patients must be residents of Norway and understand oral and written Norwegian. Only patients with reported pain intensity (NRS) of 3 or more (with a maximum of 10) during an activity at baseline will be included.

The exclusion criteria are as follows: treatment with radial shock wave therapy for the last 3 months, spondyloarthropathy or rheumatoid arthritis, plantar fibromatosis, tarsal tunnel syndrome, polyneuropathy, previous surgery with remaining osteosynthesis material in the foot or ankle and contraindications for radial shock wave therapy (use of anticoagulant drugs, pregnancy, bleeding disorders, epilepsy or pacemaker).

**Fig. 1:**
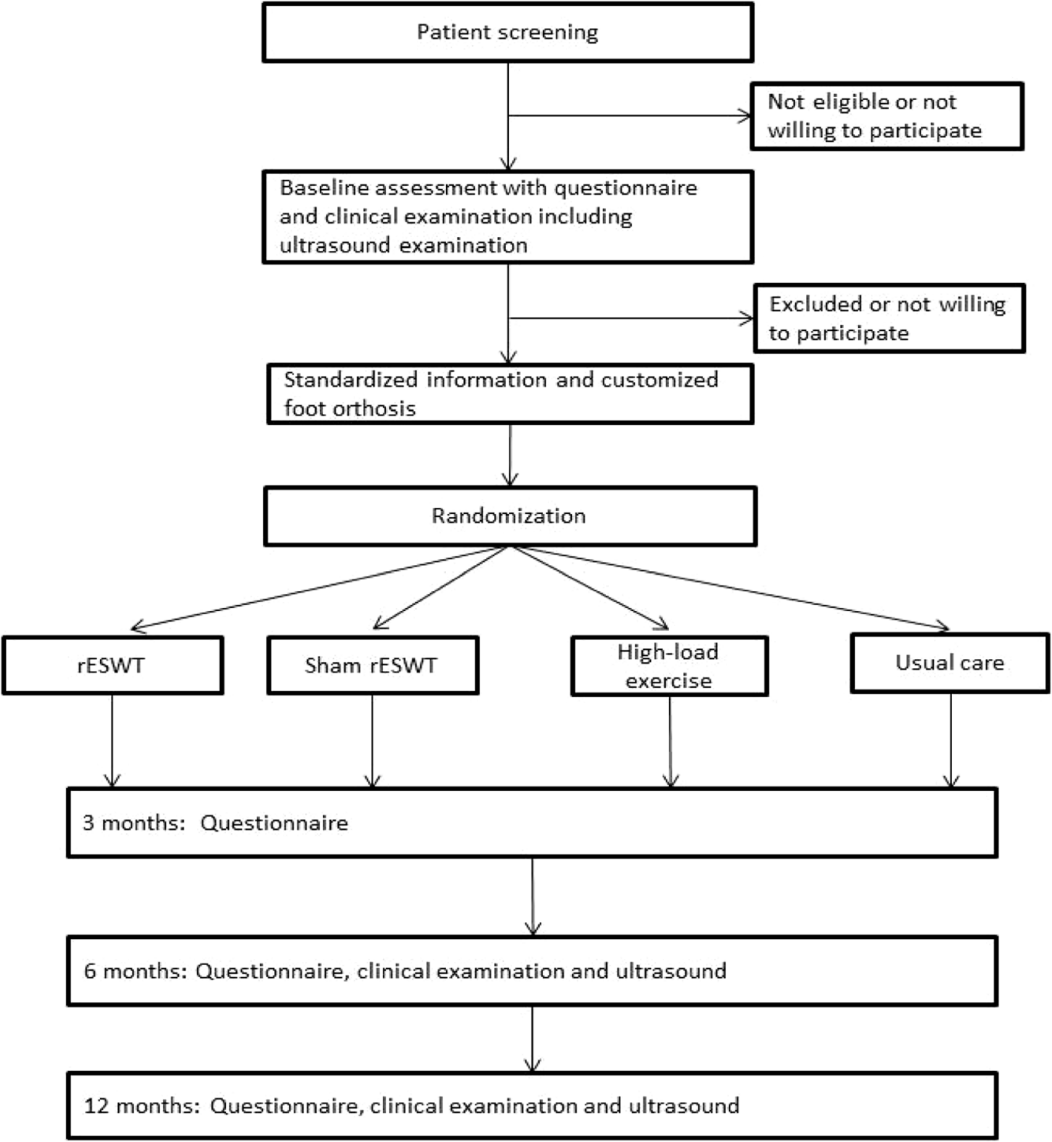
Flowchart

Patients who do not fulfil the inclusion criteria or do not want to participate in the study will be given treatment as usual at our outpatient clinic (“usual care”). Participants will be asked for permission for the research team to share relevant data with people from the university taking part in the research or from regulatory authorities, where relevant. This trial does not involve collecting biological specimens for storage (Fig. 1).

### Randomisation and blinding

Patients who fulfil the inclusion criteria will be given oral and written information about the trial by a physician (MH or AFH). The patients who give their informed consent will then be randomised into one of the four groups: rESWT, sham-rESWT, standardised exercise or usual care. A computer generation randomisation schedule with blocks of 8, in a 1:1 ratio, will be performed by an external statistician and electronically concealed. One experienced physiotherapist (MM) will serve as the project coordinator and communicate the project allocation to the patients. The patients will be blinded for rESWT/sham-rESWT, whereas blinding of the exercise and usual care group is not possible. Instructions of the exercises and follow-up of the patients in the exercise group will be performed by an experienced physiotherapist (MM). The physiotherapists providing rESWT/sham-rESWT and exercise programs will not be blinded. The outcome assessors and data analysts will be blinded to the group assignment.

To evaluate the blinding of the rESWT, the patients will be asked after the last treatment whether they believe that they have received real rESWT, sham-rESWT or if they do not know.

If severe medical events should occur, the manager of the department will have access to unblind that particular patient.

### Interventions

All patients, regardless of the treatment group, will receive standardised information from an experienced physiotherapist at our outpatient clinic (MM) before randomisation to any of the intervention groups. This information is part of our usual care on plantar fasciopathy and will be given in oral and written form as described in the introduction. All patients, regardless of the treatment group, will be referred to an orthopaedic technician who will perform a 3D scan of the foot and prepare the customised foot orthosis. The orthosis will be made of a semisolid material named Comfortline. The participants will receive instructions on using them as much as possible, indoor as well as outdoor. They will be offered one follow-up for further customization of the orthosis if necessary.

#### Radial shock wave therapy/sham-radial shock wave therapy

The patients randomised to rESWT and sham-rESWT will receive treatment or sham treatment once a week, with a maximum of 3 treatments given by a physiotherapist trained in rESWT delivery. Treatment will be administered by using the rESWT device named Swiss DolorClast (EMS). Two thousand impulses of shock waves are implied via the handpiece to the area of maximum tenderness at the insertion of the plantar fascia, with a pressure of 1.5–3 bars depending on what the patients can tolerate [20]. We will use a power handpiece which provides an energy of 0.01–0.35 mJ/mm^2^. It is known that medical procedures can arise placebo effects that mimic the effectiveness of the procedure itself, and therefore we consider it important to include the sham-rESWT group. The sham-rESWT will be administered in the exact same way as the real rESWT. The sham probe is similar in design, sound and shape, but no real shock waves will be applied.

#### Standardised high-load exercise

Patients randomised to the high-load exercise programme will be instructed to do 2 exercises: unilateral heel raise and unilateral leg squat, three times a week for 12 weeks. The patients will be instructed to do the heel raise exercise on a stairway or a similar location. As in the study of Rathleff et al. [23], the participants will have a towel or similar under their toes, ensuring that the toes are maximal dorsal flexed at the top of the heel raise. Every heel raise consists of a 3-s concentric phase/going up, a 2-s isometric phase/pause at the top and a 3-s eccentric phase/coming down [23]. As previously reported by Kongsgaard et al., the high-load exercise program will slowly progress throughout the trial [30]. We will use three sets in each exercise with 2–3-min rests between sets. The participants start with a 12 repetition maximum (RM) in weeks 1–3, 10 RM in weeks 4–6, 8 RM in weeks 7–9 and 6 RM in weeks 10–12. If they are not able to perform the required number of repetitions, they will start doing the exercise with both legs. When ready to increase the load, the participants will add load in a backpack. The unilateral leg squat will also be performed standing with a towel under their toes. The participants will balance on one leg and bend the knee over their toes in a slow movement. The exercise will strengthen the stabilising muscles of the ankle, knee and hip joints. The progression and eventual adjustments will be performed in the same manner as described in the first exercise. In the case of bilateral pain, they will be instructed to perform the exercises with both legs. The patients will have a total of 8 sessions supervised by a physiotherapist at our department during the 12 weeks to ensure the quality of performance and proper progression. The patients will also receive a one-page manual including pictures of the exercises together with a description of the progress and a training diary in which sets, repetitions, weight load and number of training sessions per week will be registered. Compliance will be examined.

#### Usual care

The usual care comprises “standardised information” (pathogenesis, aetiology and prognosis based on the latest research) and custom-made foot orthosis. The “standardised information” is given by the physiotherapist (MM) in oral and written.

### Outcome measurements and follow-up

The patients eligible for inclusion are referred to one of three experienced physicians who will examine all the patients at baseline. Patients included will be asked to complete a self-administered questionnaire at the primary consultation detailing the following baseline data: age, gender, relationship status, education, occupational status, sick leave status, duration of symptoms, previous treatments including radial shock wave treatment, use of analgesics the last 4 weeks, physical activity level and smoking habits.

It is known that the patients’ expectations can influence the outcome in clinical trials [31]. At baseline, we will ask the patients about their expectations of change in foot pain. We will use a 7-point scale ranging from “very much improved” to “very much worse”, based on the Patient Global Impression of Change Scale (PGIC) [32, 33]. We will ask the patients what intervention they prefer receiving (rESWT, exercises or usual care).

The primary outcome measure is the numeric rating scale (NRS) during activity and at the 6-month follow-up. Study outcomes (primary and secondary) and time points are summarised in Table 1.

**Table 1:** Schedule of enrolment, interventions, and assessments during the study period

	Enrolment	Allocation	Interventions	Follow-up		
Time point	0	0	0–3 months	3 months	6 months	12 months
Enrolment:						
Eligibility screen	X					
Informed consent	X					
Allocation		X				
Interventions:						
rESWT			X			
Sham-rESWT			X			
Exercise programme			X			
Usual care 1)			X			
Assessments:						
Baseline variables 2)	X					
NRS 3)	X			X	X	X
FFI-RS 4)	X			X	X	X
RAND-12	X			X	X	X
PGIC 5)				X	X	X
Other data variables 6)				X	X	X
Clinical examination and ultrasound 7)	X				X	X

1) Usual care: all patients get the same information before randomisation. 2) Baseline variables: age, gender, relationship status, education, work situation, sick leave, duration of symptoms, previous treatment, previous radial shock wave treatment, use of pain medication, physical activity, smoke/non-smoker, expectations of change in foot pain, which treatment the patient hopes he/she will get in the trial. 3) NRS: numeric rating scale.4) FFI-RS: Foot Functional Index Revised Short Version. 5) PGIC: Patient Global Impression of Change. 6) Other variables: use of foot orthosis, side effects of treatment, use of other treatment modalities, use of pain medication. 7) Clinical examination includes palpation of the ankle and foot including the insertion of the plantar fascia, weight-bearing ankle dorsiflexion motion, calf-raise test. In addition and only at inclusion: height, weight, body mass index, measurement of calf circumference, passive range of motion in the ankle joint. Ultrasound measurement includes thickness in millimetres, hypoechogenicity (presence/no presence), neovascularization (presence/no presence) and calcification (presence/no presence)

*NRS* is an 11-point scale consisting of integers from 0 to 10, with 0 representing “no pain” and 10 representing “worst imaginable pain”. NRS is a reliable and sensitive scale detecting pain [33]. The patients will be asked to assess pain during activity and rest for the previous week.

*Secondary outcomes* are Foot Functional Index short version revised (FFI-RS) at 6 and 12 months, RAND-12 Health Status Inventory (RAND-12) at 6 and 12 months, NRS in rest at 6 and 12 months, NRS in activity at 12 months and the Patient Global Impression of Change Scale (PGIC) at 6 and 12 months.

*FFI-RS* is a known, self-administered instrument measuring foot health status, which has been used in previous studies of foot and ankle disorders. It consists of 34 questions (short form) and five subscales: pain, stiffness, difficulty, activity limitations and social issues. We will use the short form for the measure because it is as reliable as the long form (68 questions), and the response burden on patients is lower [34]. The questionnaire will be translated and validated into Norwegian according to the international guidelines [35].

*RAND-12* is a commonly used self-report instrument measuring health-related quality of life. It is the much shorter alternative to the RAND-36 Health Status Inventory or SF-36. It consists of 12 items where six of them create the physical health composite, and the remaining six create the mental health composite [36].

*PGIC* is used to measure participants’ self-reported general health status, using a 7-point Likert scale ranging from “very much improved” to “very much worse” [37].

The patients will also receive a clinical examination (MH and AFH) according to a structured protocol with the registration of height, weight and body mass index, palpation of the ankle and foot including the insertion of the plantar fascia and measurement of calf circumference [38], weight-bearing ankle dorsiflexion range of motion [39], passive range of motion in the ankle joint using a gonimeter with the knee in 90° flexion and the patient in a supine position and the calf-raise test measuring endurance of the calf-muscles [40]. An ultrasound examination (MH and AFH), with a longitudinal scan of the plantar fascia, will also be performed. The thickness of the fascia at the insertion in both feet will be measured, and the average of three measurements will be used. The presence of hypoechogenicity (presence/no presence), neovascularisation (presence/no presence) and calcification (presence/no presence) will also be evaluated [11, 41]. The purpose is to evaluate whether it is an association between pain, function and ultrasound findings.

The adherence to the interventions will be recorded. All intervention groups are asked to refrain from any other treatments in the study period. We will register the use of any other treatment, the use of foot orthosis and adverse events at each follow-up. If there are bilateral symptoms, both feet will get the same treatment, but only the most painful foot at baseline will be registered. If some of the active interventions demonstrate a significant better outcome at 12 months follow-up, crossover to active treatment will be offered after 12 months.

### Statistical analysis

The sample size has been calculated based on the primary outcome measure NRS at 6 months for a comparison between two of the treatment groups using a two-sided *t* test. With a statistical test power of 90%, a significance level of 5%, an assumed difference of 2 on NRS [32], estimated standard deviation (SD) 2.7 (based on previous clinical data from the Department of Physical Medicine and Rehabilitation) and a dropout of 20%, the sample size is estimated to be 200, with 50 in each group. Patient lists will be available.

Patient characteristics, anthropometric data and the duration of symptoms in the different groups will be registered at baseline and presented as mean values (SD) for continuous variables or numbers (%) for categorical data. Pain and function scores will be presented as mean (SD). In addition to the two-group comparisons with *t* tests, we will perform a longitudinal data analysis and perform a mixed model analysis to compare the differences between the groups at follow-up with adjustment for scores at baseline and present the mean differences (95% confidence intervals).

We will perform an analysis regarding the secondary outcomes as stated above. In addition, we will apply a multivariable logistic and linear regression analysis to identify predictive factors as demographics, clinical and ultrasound findings for primary and secondary outcomes. Model building will be done in a way that is appropriate for the given sample sizes, by restricting the number of potential predictive factors and considering shrinkage methods to stabilise predictions [42].

Participants in the exercise group not attending 6 of 8 sessions with the physiotherapist or not completing 30 of 36 exercise sessions (3 sessions per week, 12 weeks) are regarded as non-adherence. In the rESWT and sham-rESWT groups, participants not attending 2 of 3 sessions are regarded as non-adherence. They will be included in the intention-to-treat analysis. There will also be a separate intention-to-treat analysis with only adherent patients.

### Implementation plan and publishing

We will follow the SPIRIT 2013 Statement (Standard Protocol Items: Recommendations for Interventional Trials) for protocol guidance to ensure transparency and a complete description of what is intended [43]. The results will be published in international referee-based scientific journals. Experiences and results from the project will also be disseminated in relevant expert forums, national meetings, conferences and reports.

## Discussion

Plantar fasciopathy is a common cause of plantar heel pain [1], and there is still no agreement on the most effective treatment for this condition [44]. Randomised controlled trials (RCT) is regarded as the gold standard in evaluating health care intervention [45]. Foot orthosis, rESWT and exercise are common conservative modalities suggested in the treatment on this condition. There is a need of more high-quality RCT’s to investigate the effectiveness of these treatments. We have included sham treatment in this study to make sure that different results in the groups (rESWT or sham-rESWT) are due to the rESWT and not placebo effects.

It has been emphasised that plantar fasciopathy is most likely a self-limiting condition [4–6]. As the patients included are referred to specialised care, a “no treatment group” is not obtainable. However, we have tried to be as close to the natural course of the disease as possible by including short and standardised information. Furthermore, our clinical experience is that foot orthosis may relieve symptoms and is also included as part of the usual care.

Standardised information and foot orthosis are included in all groups, hence obtaining the additional effect of exercises and rESWT/sham-rESWT. On the basis of the widespread use of rESWT or exercises, the results of this study will be of major interest both to the clinicians and the patients struggling with plantar fasciopathy. A positive result of some of the treatment modalities will support the practice, while no difference between the groups indicates that the use of usual care is to be recommended. Because of the nature of the condition (reduction in physical activity and health-related quality of life) in addition to the following financial burden on the community, it will be important in both a socioeconomic perspective and an individual basis to find the most cost-effective treatment for patients with plantar fasciopathy.

## Trial status

This is protocol version 3; issue date is October 28, 2019. The recruitment of the study began on March 23, 2018. The recruitment will be complete on March 23, 2021.

## Data Availability

All study-related information will be stored securely at the study site. It will be created as coded depersonalised data where the participants identifying information is replaced by an unrelated sequence of characters. A range of checks for all variables will be conducted. Data will be stored on a server at Oslo University Hospital dedicated to research. All access will be authorised by the Data Protection Officer at Oslo University Hospital, and only a minimum number of persons will get access. Information about the participants will be anonymised 5 years after the end of the trial. The datasets used and analysed during the current study are available from the corresponding author on reasonable request.

## Abbreviations

ESWT: Extracorporeal shock wave therapy
rESWT: Radial extracorporeal shock wave therapy
FFI-RS: Foot Functional Index Revised Short Version
PGIC: Patient Global Impression of Change Scale
RAND-12: RAND-12 Health Status Inventory
NRS: Numeric rating scale
RCT: Randomised controlled trial
